# High-fat diet-induced acceleration of osteoarthritis is associated with a distinct and sustained plasma metabolite signature

**DOI:** 10.1038/s41598-017-07963-6

**Published:** 2017-08-15

**Authors:** Poulami Datta, Yue Zhang, Alexa Parousis, Anirudh Sharma, Evgeny Rossomacha, Helal Endisha, Brian Wu, Izabela Kacprzak, Nizar N. Mahomed, Rajiv Gandhi, Jason S. Rockel, Mohit Kapoor

**Affiliations:** 1 Arthritis Program, University Health Network, Toronto, Ontario, Canada; 2 Krembil Research Institute, University Health Network, Toronto, Ontario, Canada; 3 Department of Laboratory Medicine and Pathobiology, University of Toronto, Toronto, Ontario Canada; 4 Department of Surgery, University of Toronto, Toronto, Ontario, Canada

## Abstract

Metabolic changes induced by high fat diet (HFD) that contribute to osteoarthritis (OA) are poorly understood. We investigated longitudinal changes to metabolites and their contribution to OA pathogenesis in response to HFD. HFD-fed mice exhibited acceleration of spontaneous age-related and surgically-induced OA compared to lean diet (LD)-fed mice. Using metabolomics, we identified that HFD-fed mice exhibited a distinct and sustained plasma metabolite signature rich in phosphatidylcholines (PC) and lysophosphatidylcholines (lysoPCs), even after resumption of normal chow diet. Using receiver operator curve analysis and prediction modelling, we showed that the concentration of these identified metabolites could efficiently predict the type of diet and OA risk with an accuracy of 93%. Further, longitudinal evaluation of knee joints of HFD- compared to LD- fed mice showed a greater percentage of leptin-positive chondrocytes. Mechanistic data showed that leptin-treated human OA chondrocytes exhibited enhanced production of lysoPCs and expression of autotaxin and catabolic MMP-13. Leptin-induced increased MMP13 expression was reversed by autotaxin inhibition. Together, this study is the first to describe a distinct and sustained HFD-induced metabolite signature. This study suggests that in addition to increased weight, identified metabolites and local leptin-signaling may also contribute in part, towards the accelerated OA-phenotype observed in HFD mice.

## Introduction

Osteoarthritis (OA) is the most common form of arthritis worldwide. It is characterised by alterations in articular cartilage and subchondral bone, osteophyte formation and inflammation of the synovium^1^. Aetiologies of this disease are poorly defined; however, epidemiological studies suggest that obese individuals have an increased incidence of OA and are 33% more likely to require joint replacement surgery than individuals with a normal body mass^2^. Globally, obesity is one of the leading risk factor for death and it predisposes individuals to many diseases including OA^3, 4^. The World Health Organization suggests an individual with body mass index (BMI) greater than or equal to 30 kg/m^2^ as being obese. This excessive and abnormal fat accumulation is an important risk factor for increasing incidence of OA by imparting increased mechanical forces across weight bearing joints^5^. Excess body fat has also been associated with OA in non-weight bearing joints (such as the hands), indicating that metabolic factors may also contribute to OA pathogenesis^6^.

Although obesity can result from excessive intake of high calorie diet and decreased physical activity, it may also occur due to dysregulated secretion of adipokines and other metabolic factors^7–11^. Interestingly, changes to metabolite levels in the blood, as a result of changes to tissue and body composition are also implicated in the pathogenesis of OA^12^. Metabolomics, the comprehensive analysis of metabolites in body fluids, cells and tissues, can help identify specific metabolite profiles that can be used as markers of pathologic disease. Altered metabolite levels are observed in obese individuals and in other metabolic disorders, in addition to dysregulated secretion of cytokines and adipokines^13–15^. Mice subjected to high fat diet (HFD) have also shown defined metabolite signatures associated with obesity^16, 17^. However, the exact longitudinal changes in the metabolites of mice subjected to HFD and how alterations in these metabolites influence the pathogenesis of OA is largely unknown. Therefore, the aim of this study was to identify longitudinal changes in the metabolite profiles resulting from HFD and LD regimes in mice and identify the role and mechanisms of their contributions to OA pathogenesis.

## Results

### High fat diet induces changes in body weight, fasting blood glucose and insulin levels

**Figure 1:**
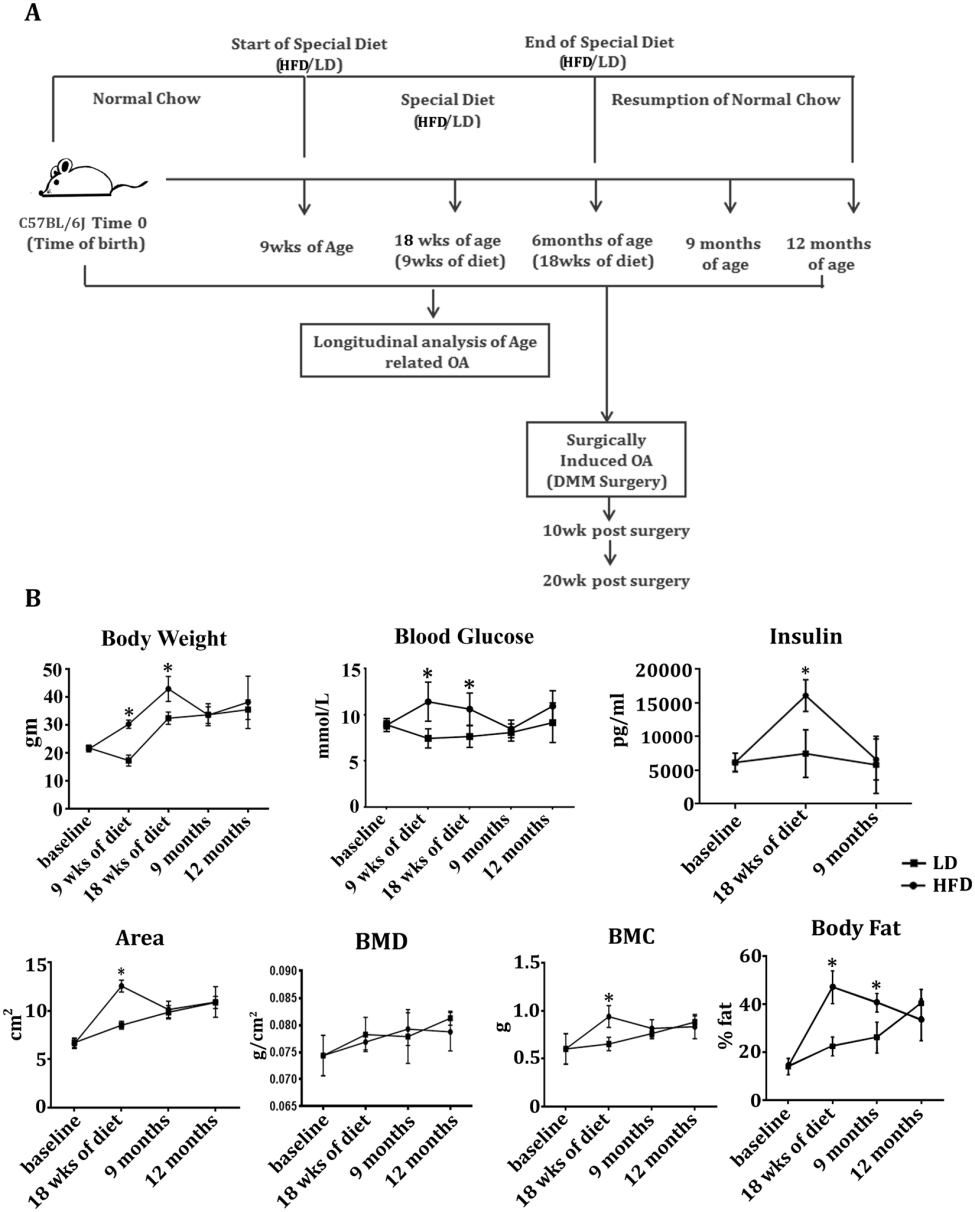
High fat diet promotes weight gain, hyperglycemia, hyperinsulemia and changes to body composition. (**A**) Schematic diagram showing diet regimes of high fat diet- (HFD), lean diet- (LD) and normal chow-fed mice, and the longitudinal timing for analysis of age related and surgically-induced OA. (**B**) Nine-week old C57BL/6J mice were placed on high fat diet or lean diet until 18 weeks (6 months of age) at which time the mice were returned to a normal chow diet up to 12 months of age. Body weight, fasting blood glucose, insulin levels, body area, bone mineral density (BMD), bone mineral content (BMC) and body fat percent were determined longitudinally. All results are expressed as mean ± SD. Data were analyzed by Two-way ANOVA followed by Holm-Sidak multiple comparison tests. *p < 0.05 (LD vs HFD at each time point); n = 10.

To initially determine the effect of diet on physical parameters and blood chemistry, 9-week old C57BL/6J mice were placed on either HFD or LD for 18 weeks (Fig. 1A). After 9 weeks and 18 weeks of diet, HFD-fed mice showed a significant increase in body weight and fasting blood glucose compared to LD-fed mice (Fig. 1B). After completion of special diet regimes and resumption of normal chow, mice at 9 months and 12 months of age exhibited no significant differences in the body weight or fasting blood glucose between the HFD and LD-fed groups. HFD-fed mice also showed increased insulin levels at 18 weeks of diet as compared to LD-fed mice (Fig. 1B); however, insulin levels were not significanly different in HFD-fed mice as compared to LD-fed mice after resumption of normal chow at 9 months and 12 months of age.

To identify longitudinal changes to body composition, we measured body area, body fat percentage, bone mineral content, and bone mineral density by DEXA. Mice on HFD had significantly increased body area, percent of body fat and bone mineral content at 18 weeks of diet as compared to LD-fed mice. Bone mineral density showed no significant change between groups. After resumption of normal chow, only body fat remained significantly elevated at 9 months of age, and resolved to a similar level as LD-fed mice by 12 months of age (Fig. 1B and Supplementary Fig. 1).

### High fat diet-fed mice exhibit accelerated surgically-induced OA

**Figure 2:**
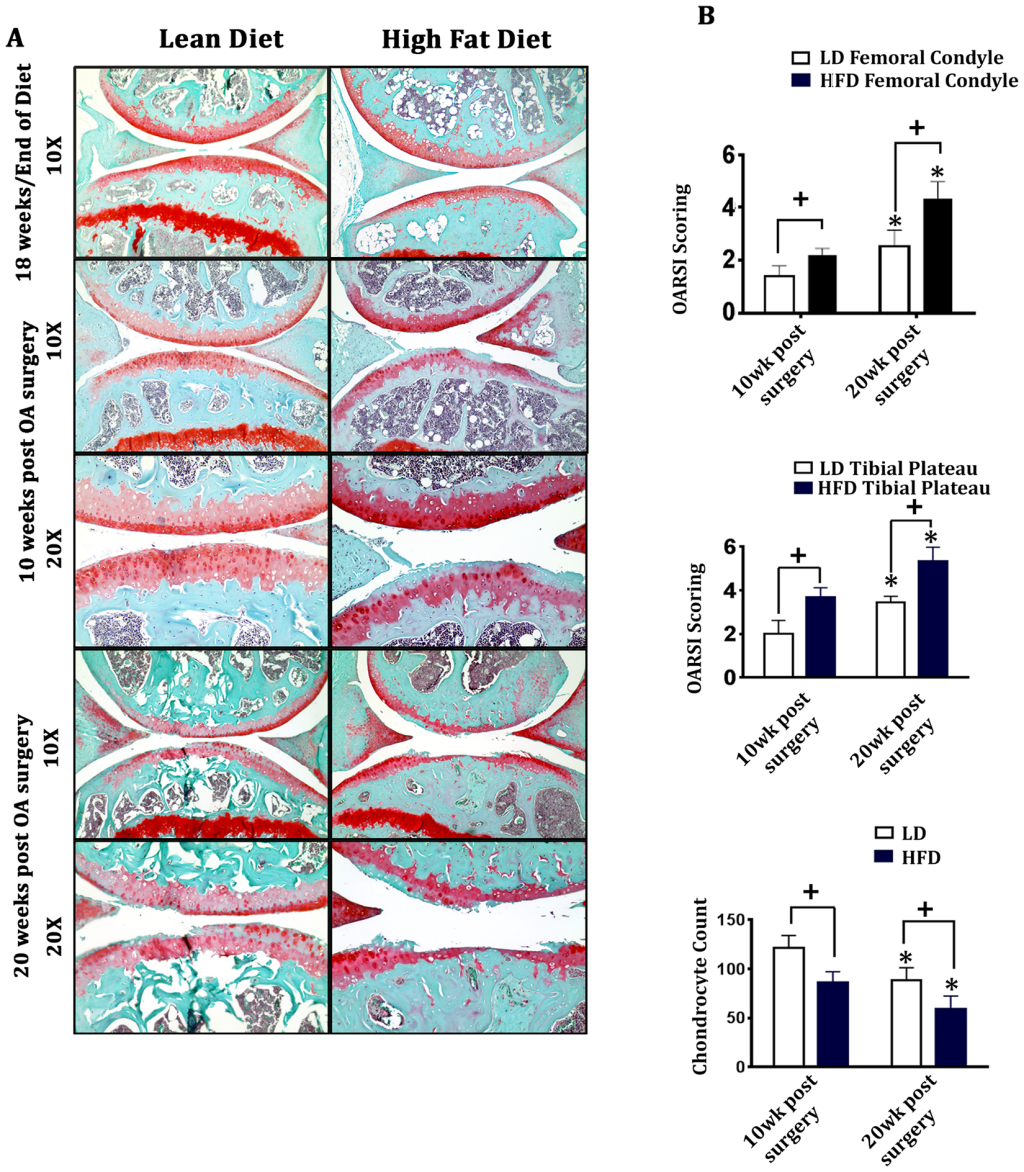
High fat diet-fed mice exhibit accelerated surgically-induced OA. (**A**) Representative histological images of Safranin-O/Fast Green stained knee joint sections from lean diet (LD) and high fat diet (HFD)-fed mice that underwent destabilisation of medial meniscus (DMM surgery) at 18 weeks of diet (6 months of age) and were collected at 10 or 20-weeks post-surgery. Magnification is 10x and 20x. (**B**) Severity of OA pathogenesis was determined by OARSI histopathology grading of mouse femoral condyles and tibial plateaus, and number of chondrocytes per area were counted. Data were analysed by Two-way ANOVA followed by Holm-Sidak multiple comparison tests. Data are expressed as mean ± SD (n = 10). ^+^p < 0.05, LD vs HFD at 10-weeks or LD vs HFD 20-weeks post-surgery. *p < 0.05, HFD at 10-weeks vs HFD at 20-weeks of post-surgery or LD at 10 weeks vs LD 20 weeks of post-surgery.

Next, we determined the effect of HFD (compared to LD) on knee articular cartilage integrity (medial tibial plateau and medial femoral condyle) in mice subjected to DMM (destabilisation of medial meniscus) surgery. At 18 weeks of diet, HFD-fed mice showed slight roughening of the articular cartilage as compared to LD-fed mice with no other profound histomorphometric changes to the articular cartilage (Fig. 2A). In surgically-induced OA mice, a progressive worsening of OA was observed between 10 weeks and 20 weeks post-surgery in both HFD and LD-fed mice (Fig. 2A); however, OA was more severe in HFD-fed mice compared to LD-fed mice at both 10 and 20 weeks post-surgery, as determined by Osteoarthritis Research Society International (OARSI) scoring (Fig. 2B). Both diet groups showed decreased chondrocyte cellularity between 10 and 20 weeks post-surgery; however loss of chondrocyte cellularity was significantly greater in HFD-fed mice compared to LD-fed mice.

### High fat diet-fed mice exhibit accelerated age-related spontaneous OA

**Figure 3:**
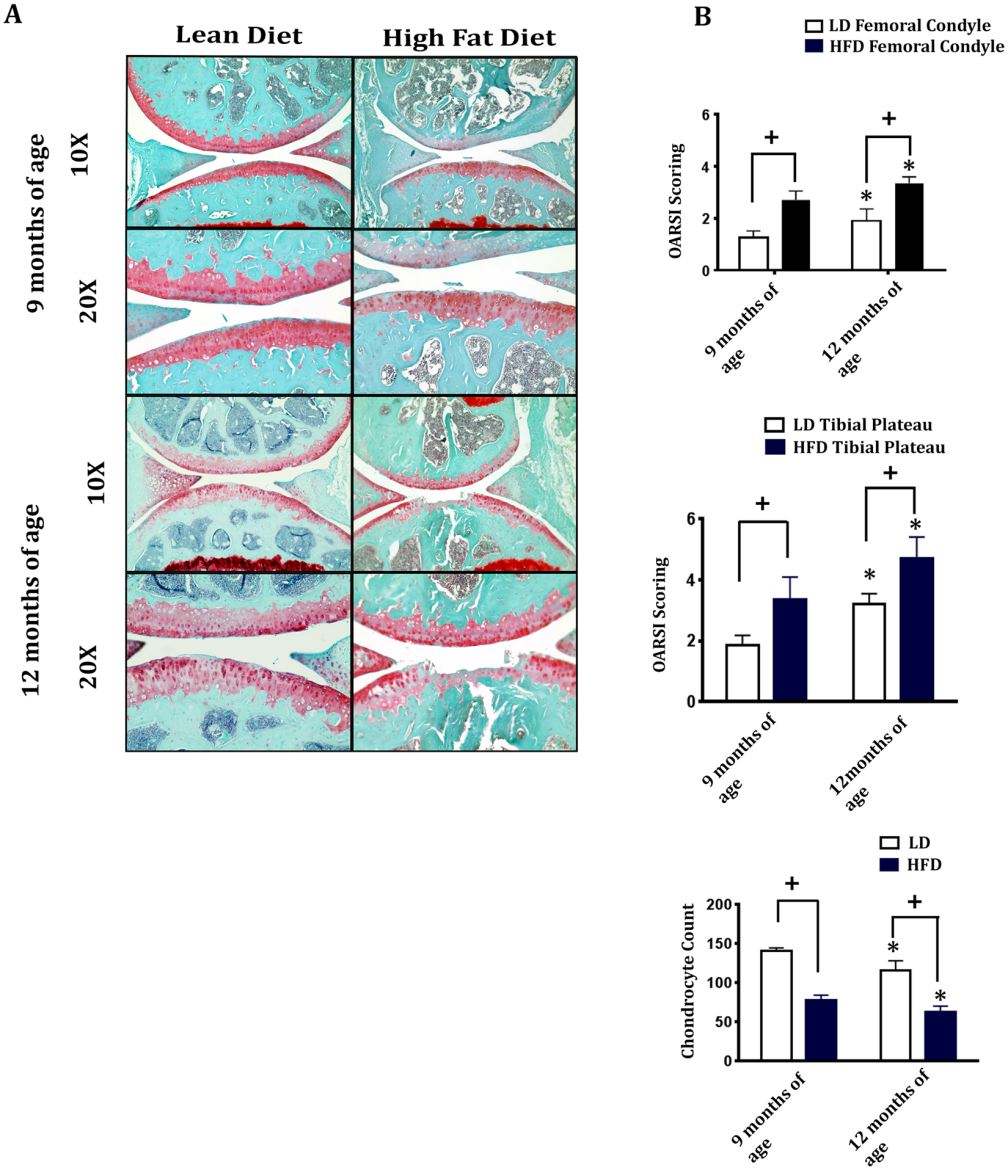
High fat diet-fed mice exhibit accelerated spontaneous OA. (**A**) Representative histological sections of Safranin-O/Fast Green stained knee joints from lean diet (LD) and high fat diet (HFD)-fed mice at 9 months and 12 months of age. Magnifications: 10x and 20x. (**B**) Severity of OA pathogenesis was determined by OARSI histopathology grading of mouse femoral condyles and tibial plateaus and number of chondrocytes per area were counted. Data are expressed as mean ± SD. Data were analysed by Two-way ANOVA followed by Holm-Sidak multiple comparison tests. */^+^p < 0.05; n = 10. ^+^p < 0.05 LD vs HFD at 9 months of age LD vs HFD at 12 months of age. *p < 0.05 HFD at 9 months of age vs HFD at 12 months of age and LD at 9 months of age vs LD at 12 months of age (n = 10).

We next determined the effect of HFD on articular cartilage integrity in mice at 9 months and 12 months of age. As observed in surgically-induced OA model, HFD-fed mice exhibited accelerated cartilage degeneration at 9 months and 12 months of age compared to LD- fed mice as demonstrated by histomorphometry and OARSI scoring (Fig. 3A and B). Furthermore, chondrocyte loss was greater in HFD-fed mice compared to LD-fed mice at 9 months and 12 months of age. As a control, we also compared mice fed a diet exclusively of normal chow throughout the time course compared to mice that were on the LD regime. Normal chow diet-fed mice showed no significant difference in articular cartilage degeneration compared to LD-fed mice at 9 months or 12 months of age as determined by OARSI scoring (Supplementary Fig. 2A,B). Also, there was no difference in body weight between LD-fed mice and normal chow-fed mice between 18 weeks of diet, 9 months and 12 months of age (data not shown).

We also investigated if distinct diet regimes exhibited any effect on the degree of synovitis in spontaneous and surgery-induced OA. No significant difference in the degree of synovitis was observed in mice at 12 months of age or at 20 weeks post surgery in all diet groups (Supplementary Fig. 2B).

### High fat diet fed mice exhibit a distinct, longitudinal metabolite profile

**Figure 4:**
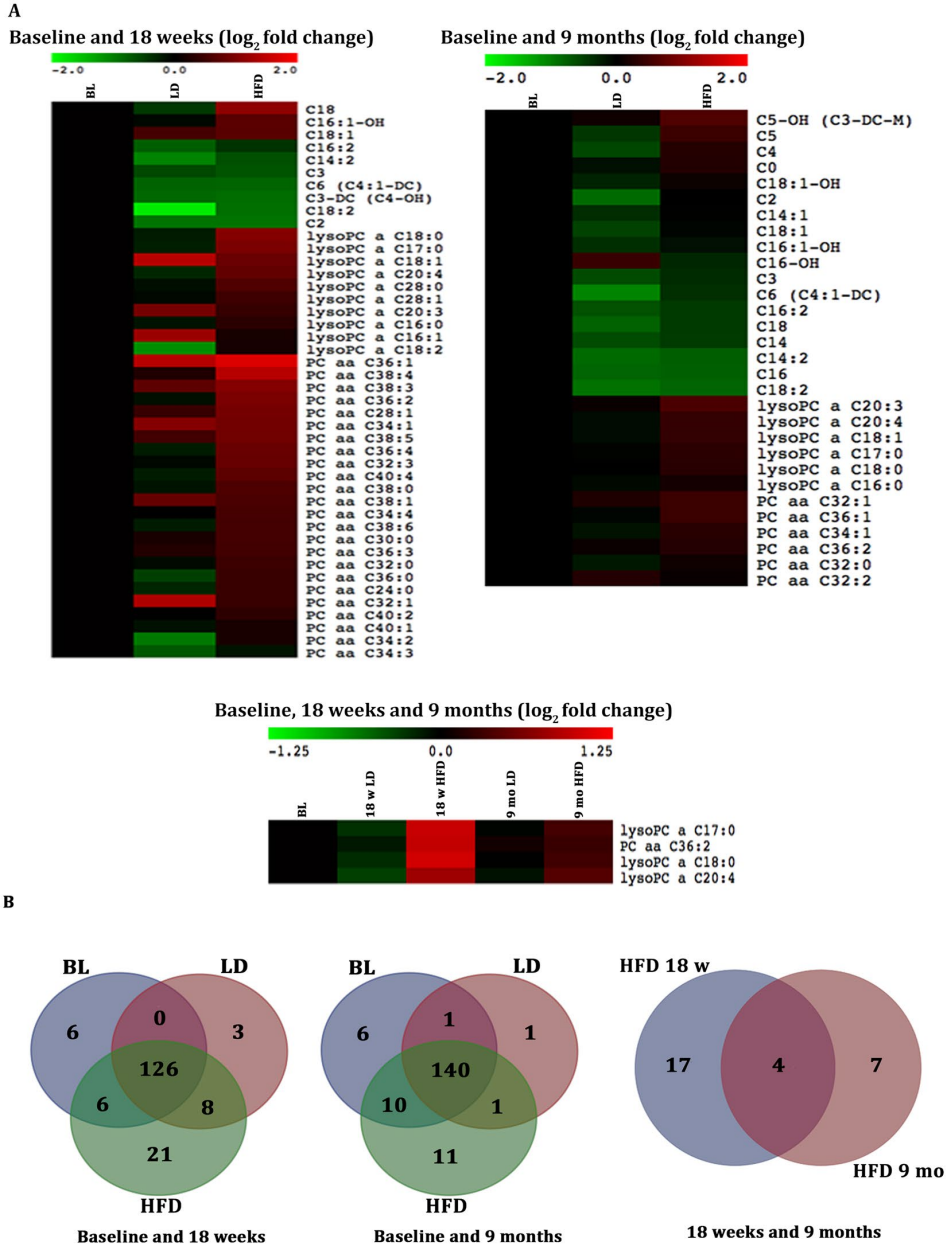
High fat diet-fed mice exhibit distinct and sustained longitudinal changes in selected metabolites. Metabolites shown were selected based on VIP scores ≥1 and One-way ANOVA (p < 0.05). (**A**) Heat maps showing the median-normalized fold change in the metabolites levels of mice at baseline, and 18 weeks of high fat diet (HFD) or lean diet (LD), or at baseline and 9 months of age from HFD- and LD- fed mice. (**B**) Venn diagrams representing metabolites identified and grouped based on One-Way ANOVA and Fisher's LSD post-hoc tests (p < 0.05 representing a significant difference between individual groups). Metabolites not meeting predetermined cut-offs are located centrally in the 18 week and 9 month venn diagrams (126 and 140 metabolites, respectively) (n = 10 per group per time point).

To determine longitudinal changes in metabolite profiles of mice on HFD and LD regimes, plasma metabolite levels (metabolomics) were assessed for each group at baseline as controls (9 weeks of age; before start of special diet regimes), 18 weeks of diet and 9 months of age. A total of 170 metabolites (21 amino acids, 14 biogenic amines, 30 acylcarnitines, 89 phosphatidylcholines, 15 sphingolipids and hexose) were identified & quantified by direct injection liquid chromatography mass spectrometry (MS)/MS. An unsupervised multivariate principal component analysis (PCA) was performed to visualize the distribution of the sample variances and variable importance in projection (VIP) scores were used to represent the importance of the particular metabolites in discriminating the groups of interest. Metabolites having VIP scores ≥ 1 that were also significantly different between groups by ANOVA (p < 0.05) were considered for further analysis (Fig. 4A). At 18 weeks (end of diet) and 9 months of age (after resumption of normal chow diet for 3 months), 21 and 11 metabolites, respectively, were exclusively upregulated in HFD-fed mice compared to LD-fed and normal chow-fed mice (Fig. 4B). Interestingly, 4 metabolites remained elevated after resumption of normal chow diet at 9 months of age exclusively in HFD-fed mice. At 18 weeks of diet and 9 months of age, there were 3 and 1 metabolite(s), respectively, that were exclusively upregulated in the LD-fed mice; however, metabolites identified at 18 weeks of diet were different than those found at 9 months of age, indicating no maintenance of the levels of these metabolites with resumption of a normal chow diet (Fig. 4B).

Of the 4 metabolites longitudinally and exclusively upregulated in HFD-fed mice, three were lysophophatidylcholine (lysoPC) analogues (lysoPCaC20:4, lysoPCaC17:0, lysoPCaC18:0) and one phosphatidylcholine (PC) analogue (PCaaC36:2). Thus, from our metabolomics studies, there appears to be a lasting alteration in metabolic pathways affecting lipid metabolism that may influence cartilage destruction in response to HFD.

### Metabolite concentrations can predict diet and likely OA severity

To assess the probability of these metabolites to identify the severity of OA in the knee joints﻿ of HFD- and LD- fed mice﻿, we utilized matched plasma and OARSI scores from 9-months of age HFD- and LD-fed mice. Groups were separated depending on tibial plateau OARSI scores <3 (non-severe) or ≥3 (severe) (n = 10 total). Univariate receiver operator curves (ROCs) were generated and the area under the curve (AUC) as well as t-tests of the non-severe vs severe groups were determined (Table 1) followed by generation of multivariate ROCs using random forest classification modeling (Table 2). We determined that using 3 metabolites, namely lysoPCaC20:4, lysoPCaC17:0 and PCaaC36:2, which had the highest AUCs and lowest p values as determined by univariate ROC analysis and t-tests, respectively (Table 1), resulted in a classification model with a higher AUC and model accuracy compared to the classification model generated using all 4 identified metabolites (Table 2). Using the classification model from the top 3 metabolites, we found that from the data used to generate the model, two mice were classified into categories opposite to those determined by OARSI scoring (Supplementary Table 1). However, of the remaining plasma samples analyzed for metabolomics analysis, which included plasma samples from HFD- and LD-fed mice at 18 weeks of diet and 9 months of age (n = 29), only two HFD-fed mice were predicted to have a non-severe phenotype [93% overall rate of matching diet to predicted OA outcome based on study observations (Fig. 3 and Supplementary Table 2)], with all other mice matching severe OA to HFD-fed mice and non-severe to LD-fed mice. Overall these data suggest the potential of lysoPCaC20:4, lysoPCaC17:0 and PCaaC36:2 to predict OA risk, incidence or severity, without knowing prior dietary regimes.

**Table 1:** ROC analysis of metabolites longitudinally upregulated in HFD-induced osteoarthritis.

Metabolite	AUC	95% CI	Fold change (HFD relative to LD)	T-test
lysoPCaC17:0	0.96429	0.786–1	1.40	0.0035
lysoPCaC20:4	0.92857	0.714–1	1.60	0.0063
PCaaC36:2	0.92857	0.714–1	1.20	0.0176
lysoPCaC18:0	0.85714	0.571–1	1.28	0.0419

Table of selected metabolites identified from VIP ≥ 1 and significantly upregulated by One-way ANOVA (p < 0.05) at 18 weeks of diet and 9 months of age in HFD-fed mouse plasma vs LD-fed or baseline mouse plasma. AUC; area under curve, CI; confidence interval; HFD; high fat diet; LD; Lean diet. Significant P-values (p < 0.05) determined by Student’s t-test of log-transformed, auto-scaled data comparing animals with severe (OARSI score ≥ 3) vs. non-severe (OARSI score <3) osteoarthritis.

**Table 2:** AUC of combined metabolites of the top 3 or 4 metabolites from Table 1 based on lowest p-value and highest AUC, as determined by random forests classification modeling.

Grouped ROC	AUC	95% CI	The average accuracy based on 100 cross validations
Top 3	0.965	0.75–1	0.850
Top 4	0.92	0.5–1	0.808

ROC, receiver operator curve; AUC, area under curve; CI, Confidence interval.

### Leptin is elevated in mouse plasma and articular cartilage in response to HFD

**Figure 5:**
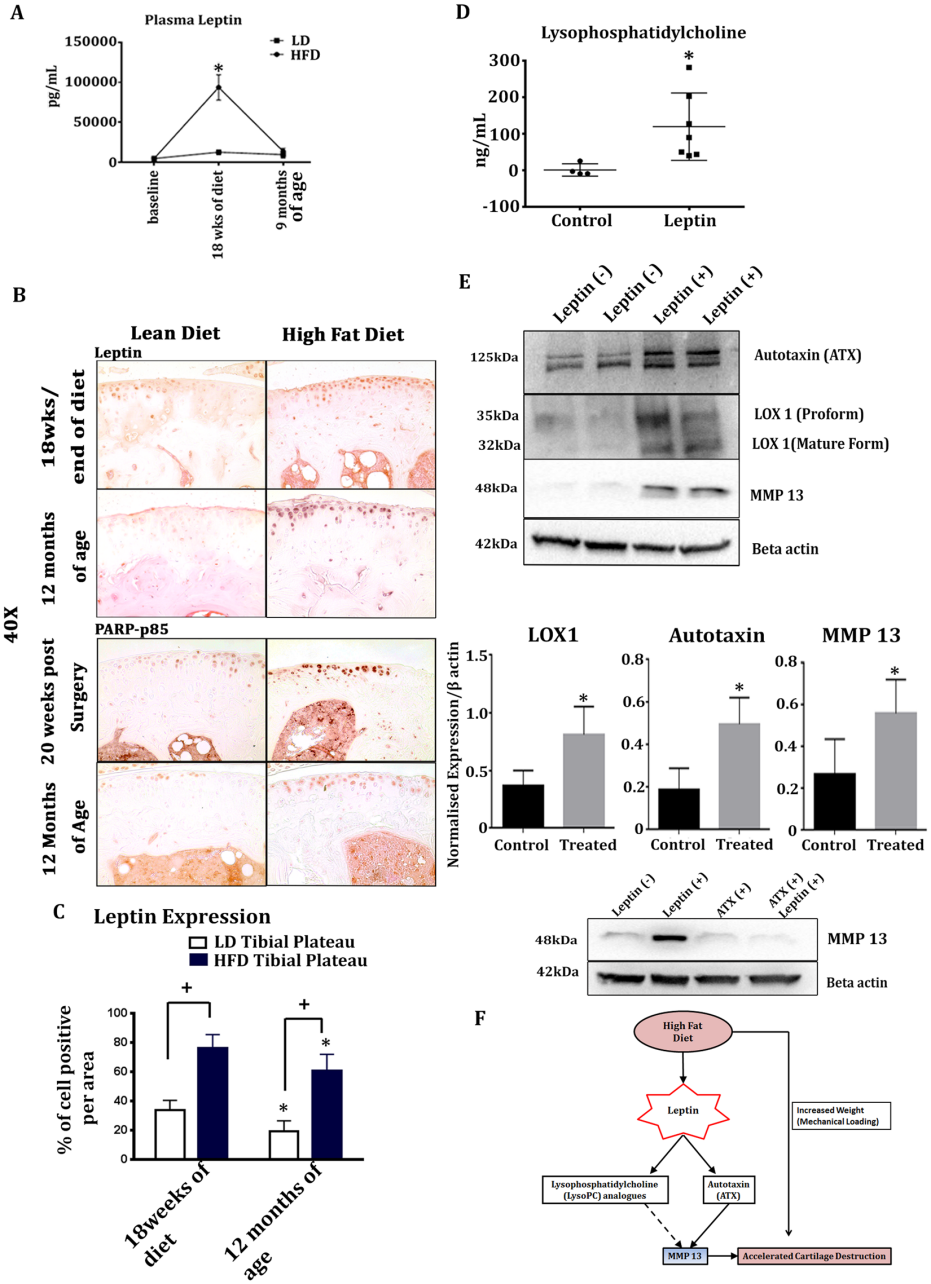
Leptin is increased longitudinally in articular cartilage but not plasma of HFD-fed mice. (**A**) Plasma levels of leptin measured by ELISA kit in HFD- and LD-fed mice at 18-weeks of diet and 9 months of age. Data are expressed mean ± SD. Data were analysed by Two-way ANOVA followed by Holm-Sidak multiple comparison tests. *p < 0.05 (LD vs HFD at 18-weeks of age) n = 10. (**B**) Leptin and PARP p85 expression in articular chondrocytes of HFD-fed mice at 18-weeks of diet, 12 months of age, or 20-weeks post-surgery compared to LD-fed mice. Images are representative of n = 5 for each diet group at each collection timepoint. (**C**) Percent of leptin positive cells/area at 18-weeks of diet (HFD or LD) and at 12 months of age. Data are expressed mean ± SD. Data was analysed by Two-way ANOVA followed by Holm-Sidak multiple comparison tests. */^+^p < 0.05; n = 5. ^+^p < 0.05 LD vs HFD at 18-weeks of diet or LD vs. HFD at 12 months of age, *p < 0.05 HFD at 18-weeks of diet vs HFD at 12 months of age and LD at 18 weeks of diet vs LD at 12 months of age. (**D**) Lysophosphatidylcholine ELISA of media supernatants of human OA chondrocytes treated with leptin. Results are expressed as mean ± SD. *p < 0.05, as analysed by two-tailed Student’s t-test. n = 11 media samples from independent wells containing chondrocytes from n = 5 independent patient samples. (**E**) Western blots showing expression of autotaxin, LOX1, MMP13 in the chondrocytes treated with leptin. β-actin was used as a loading control. Densitometry graphs of LOX1, autotaxin (ATX) and MMP13 normalised to the Beta-actin expression. Data are expressed as mean ± SD and analyzed by paired Student’s t-tests (two-tailed). */^+^p < 0.05 (n = 5). Representative western blot showing effect of autotaxin inhibitor on Leptin-induced MMP13 in human OA chondrocytes. Beta-actin expression was used as a loading control (n = 3). (**F**) Schematic showing that in addition to increased weight, local leptin signaling may contribute to the accelerated progression of OA in response to high fat diet. Solid lines indicate direct contributions. Dotted lines indicate likely but unconfirmed contributions.

After identification of the distinct metabolites longitudinally upregulated in our HFD-fed mice, we measured the level of leptin in mouse plasma, a known metabolism-regulating hormone induced by HFD and linked to OA pathogenesis (Fig. 5A)^1^. HFD-fed mice at 18 weeks of diet showed a significant increase in the level of leptin as compared to LD-fed mice. Conversely, after resumption of normal chow diet, no significant difference in the levels of plasma leptin were observed between the HFD-fed mice and LD-fed mice at 9 months of age; however, the percentage of leptin-positive articular chondrocytes in knee joints was significantly increased longitudinally in HFD-fed mice at 18 weeks of diet and 12months of age compared to LD-fed mice at the same time points, as determined by immunohistochemistry (Fig. 5B,C).

Immunohistochemical studies of PARP p85, a known apoptotic marker^18, 19^ showed increased expression in HFD- fed mice articular chondrocytes at 20 weeks post-DMM surgery and 12 months of age as compared to LD-fed mice controls from spontaneuous and DMM-surgery models, indicating accelerated loss of the chondrocytes with HFD (Fig. 5B).

### Leptin may in part contribute to metabolic and catabolic changes in articular cartilage

Since we observed increased expression of leptin locally in the articular cartilage of HFD-fed mice, we next tested if leptin may contribute towards metabolic and catabolic changes in the articular cartilage, contributing to its degeneration. To test this, we treated human OA chondrocytes *in vitro* with leptin and first determined the changes to the excretion of total lysoPCs into the media by ELISA. In response to leptin treatment, release of total lysoPCs into the media of chondrocytes in culture was increased as compared to media from vehicle-treated cultures (Fig. 5D). To confirm the response of chondrocytes to leptin we determined the expression of LOX1, a downstream target of leptin signaling^20^. We found that LOX1 was indeed significantly elevated in leptin-treated chondrocyte cultures compared to vehicle treated cultures (Fig. 5E). We then determined the expression of autotaxin (ATX), the major metabolic enzyme that converts lysophosphatidylcholine analogues to the proinflammatory mediator lysophosphatidic acid (LPA)^21^ and the expression of matrix metalloproteinase 13 (MMP13), the major catabolic enzyme responsible for cleaving type II collagen in cartilage^22^. We found that ATX and MMP13 were significantly increased in leptin treated chondrocyte cultures compared to vehicle treated cultures (Fig. 5E). We then determined the effect of leptin treatment on the expression of PARP p85 in OA chondrocytes; however, we did not observe any differences in the level of PARP p85 expression in response to leptin treatment (data not shown). We then investigated if inhibition of ATX exhibited any effect on leptin-induced MMP13 expression. Interestingly, treatment of chondrocytes with ATX inhibitor markedly decreased leptin-induced MMP13 expression (Fig. 5E). Overall, this data suggests that leptin may contribute to the local release of total lysoPC analogues and increased autotaxin expression, which, in turn, promotes cartilage degeneration by enhancing catabolic enzyme expression (Fig. 5F).

We also attempted to determine the levels of LPA in leptin-treated and/or ATX inhibitor-treated human chondrocytes using an LPA ELISA, however, we were unable to detect LPA in the media, possibly due to a high pericellular concentration but low overall concentration in the media combined with a short half-life of LPA in media (~2 h for 1 uM)^23^.

## Discussion

Osteoarthritis and obesity are two prevalent health problems worldwide with OA conventionally associated with age-related wear and tear of joints and obesity predominantly determined by diet and lifestyle^24^. Evidence has pointed to a significant role of obesity in the pathogenesis of OA^25–28^. In humans, there is a high incidence of OA in obese individuals and a strong correlation between BMI and OA has been reported^29–32^. In addition to increased mechanical loading, dysregulated secretion of certain metabolic factors may also contribute to joint degeneration during OA. In this study we subjected mice to HFD and LD to identify longitudinal changes in the metabolite profiles resulting from different diet regimes and identify the role and mechanisms of and their contributions to OA pathogenesis.

In our longitudinal study, HFD-fed showed increased body weight, body area, percent of body fat, bone mineral content, fasting blood glucose levels and increased insulin levels at 18 weeks of diet compared to LD-fed mice. After resumption of normal chow, only body fat remained elevated at 9 months of age and resolved to a similar level as LD-fed mice by 12 months of age. In contrast, we found that HFD accelerates the progression of OA in both spontaneous and surgically induced OA models. These data suggest that weight may play a crucial role in the induction of OA. However, other biological mediators may also be involved in the acceleration and progression of OA observed in HFD-fed mice. Indeed, we found that even though many measures of body size and metabolic status return to normal following a change from HFD to normal chow diet, we specifically identified that there were sustained, longitudinal increases in selected metabolites, including lysoPCaC20:4, lysoPCaC17:0, lysoPCaC18:0 and PCaaC36:2. In addition, using all of these metabolites except for lysoPCaC18:0 as biomarkers, we were able to accurately predict diet and likely cartilage degeneration by multivariate predictive classification modeling, suggesting that concentrations of selected metabolites could be used as potential biomarkers of OA disease induced by diet.

Interestingly, in a randomized control trial, obese patients placed onto a defined diet, although losing more weight compared to patients on a healthy lifestyle control, continued to show a similar progression of OA compared to the control group, suggesting that diet alone cannot attenuate OA progression^33^. Furthermore, individuals who lost a significant amount of weight due to aggressive diet and exercise showed a significant metabolic adaptation to the weight loss, needing to consume less calories than would be predicted based on final body composition and age^34^. In addition, these individuals had a persistent and more profound metabolic adaptation at 6-year follow-up after the end of the diet and exercise regime, suggesting a longitudinal adaptation, consistent to the longitudinal changes in metabolite levels observed in our study. Taken together, longitudinal adaptations to high-fat diet, including those to selected metabolites, are evident in both humans and mice and could thus play a contributing role to acceleration of OA.

Eight metabolites have previously been identified to have a significant correlation between blood plasma and synovial fluid in a human cohort of patients with OA^35^. In our study, we specifically found three lysoPCs and one PC analogue were longitudinally upregulated in the plasma of HFD-fed mice compared to LD-fed and baseline-fed mice from end of diet (18 weeks) to 9 months of age. Ratios of metabolite concentrations within blood plasma, within synovial fluid, or between blood plasma and synovial fluid have also been shown to be linked to OA. Specifically, ratios of 70 lysoPC:PC isoforms show strong and significant correlation between plasma and synovial fluid whereas the total lysoPC:PC ratio can predict advanced knee OA, suggesting that enzymatic degradation of PCs to lysoPCs are likely linked locally and systemically^35, 36^. Our data further suggests that direct metabolite concentrations rather than ratios can accurately predict OA degeneration status induced by high-fat diet.

In our studies, we observed longitudinally increased levels of selected lysoPCs and PCs in plasma of HFD-fed mice at 9-months of age, however, the levels of leptin in HFD-fed mice returned to the level of LD-fed mice by 9-months of age, which were increased at end of diet. At this point, we do not know the exact origin of leptin and mechanism associated with the increased and sustained levels of metabolites or their relationships with leptin signaling in blood. However, our *in vitro* results indicated that OA chondrocytes treated with leptin show increased lysoPC production, suggesting that leptin may in part contribute to lysoPC production locally. With regards to systemic increase in metabolites, one could speculate several possibilities. Firstly, in addition to joint cells, local expression of leptin in other body tissues/cells may also elevate in response to HFD. This combined local leptin signalling may only elevate local metabolite levels or allow them to enter the systemic circulation, contributing to the longitudinal increases in metabolite levels. Secondly, the systemic production of metabolites may be regulated independent of leptin signaling.

In support of a local-acting, longitudinal mechanism for leptin-induced lysoPC levels, leptin injected into rat joints also induces leptin protein expression in articular cartilage, suggesting a positive feedback loop that could promote further cartilage destruction^37^. Consistent with our *in vitro* observations, MMP13 is increased in human chondrocytes treated with leptin^38, 39^. Furthermore, we determined that Leptin-induced MMP13 expression in human chondrocytes *in vitro* was dependent on the coordinately increased expression and activity of ATX, as determined by our Leptin-induced ATX inhibition studies. Overall, this suggests that leptin may in part regulate the production of selected metabolites and the expression of ATX and MMP13 in chondrocytes, which can contribute to OA pathogenesis (Fig. 5F). Thus, local blockade of ATX enzymatic functions could therefore prevent further destruction in OA^40^.

Leptin treatment of chondrocytes induces chondrocyte apoptosis through reductions in autophagy, decreases in the expression of dual specificity protein phosphatase 19 and increases in Jak2/Stat3 signaling in models of OA^41, 42^. Our *in vivo* immunohistochemical studies also showed increased leptin and PARPp85 expression in articular chondrocytes of mice fed HFD compared to LD in both spontaneous and DMM-induced models of OA. However, our *in vitro* studies did not indicate changes in PARPp85 levels with 24 h leptin treatment, suggesting that leptin may contribute over time to chondrocyte cell death *in vivo* while *in vitro*, apoptosis is not immediately induced upon leptin stimulation.

Collectively, our data suggests that, in addition to increased weight, longitudinal changes to metabolite levels and local leptin signaling, in part, may contribute to the accelerated progression of OA observed with HFD. Not only have we determined the value of using systemic concentrations of specific metabolites to identify the risk of developing or the severity of OA, we have also identified a mechanism by which HFD induces longitudinal increases of leptin in local cartilage tissue that may influence the progression of cartilage degeneration. Investigating novel therapies that modulate this pathway may help to break the cycle of cartilage degeneration observed in OA and potentially attenuate or reverse OA progression.

## Materials And Methods

### Animals, Diet Regime and Surgery

C57BL/6J male mice (Jackson Laboratories, USA) were bred and housed in a 21 ± 1 °C controlled room under a 12 hr light-dark cycle at the Krembil Research Institute animal facility. Male mice were used for all *in vivo* experiments as they develop better OA characteristics than female mice, as previously reported^43^. Animals had free access to food and water. All animal studies have been performed according to relevant guidelines and regulations approved by the animal care committee of the Krembil Research Institute (Animal Protocol Number: 3729.7). Mice were placed on a normal chow (Teklad, LM-485, Harlan) from time of birth (time 0) until 9 weeks of age. At 9 weeks of age, mice were placed on either high fat diet (HFD; 34.3% fat by weight, Harlan, TD 06414) or lean fat diet (LD; 4.2% fat by weight, Harlan, TD 06416) until 18 weeks (6 months of age) at which time they were returned to a normal chow diet (5.8% fat by weight) up to 12 months of age (Fig. 1A). At the end of diet (HFD or LD regime), some mice were subjected to destabilisation of medial meniscus (DMM)^43, 44^ surgery and were maintained on a normal chow diet until 10 or 20 weeks post-surgery. For control, mice only on normal chow diet (n = 5) were maintained up to 12 months of age and knee joints were collected at 9 months of age and 12 months of age^45^.

### Measurement of Body Weight, Body Composition and Blood Glucose

Body weight of the mice was monitored longitudinally at 9 weeks of age, 18 weeks of diet (6 months of age), 9 months of age and 12 months of age using a digital weight machine (Ohaus Corporation, CS 200, USA). For whole body composition analysis, dual energy X-ray absorptiometry scanning (DEXA; tube peak potential: 80, 100, 140 kVp, Discovery A, Hologic, ﻿USA) was used. Body fat percentage, body area, bone mineral content and bone mineral density were determined at 9 weeks of age, 18 weeks of diet, 9 months of age and 12 months of age. Data was analysed using Apex 3.4 software (Hologic, USA). To measure blood glucose, mice were fasted for 6 h and blood glucose was measured using tail vein blood samples at 9 weeks of age, 18 weeks of diet, 9 months of age and 12 months of age with a One touch glucose meter (Accu-chek Aviva Model, USA).

### Plasma collection and Leptin/Insulin Analysis

Blood samples from the saphenous vein of non-anesthetized mice were collected at 9 weeks of age, 18 weeks of diet, 9 months of age and 12 months of age, in EDTA K+ coated microvettes (Sarstedt, Germany). Plasma was separated by centrifuging the blood samples at 10,000 rcf at 4 °C. Leptin and insulin levels were measured by using a Bio-Plex MAGPIX reader and Bio-Plex Manager software (Bio-Rad Laboratories, Inc., USA). Bio-Plex Pro Mouse ELISA assay kit﻿ specific for leptin and insulin (Bio-Rad Laboratories, Inc., USA) specific to mouse leptin and insulin were used according to manufacturer’s instructions. For mouse samples, plasma leptin and insulin levels were measured in duplicates for n = 10 mice per/diet/per time point

### Metabolomic Analysis

Metabolites in mouse plasma were isolated using a combination of a direct injection mass spectrometry kit (Absolute*IDQ* ^TM^ P180 Kit, Biocrates Life Sciences AG, Austria) followed by a reverse-phase liquid chromatography-MS/MS kit, following manufacturers’ instructions for targeted identification and quantification of a large number of endogenous metabolites including amino acids, acylcarnitines, glycerophospholipids, sphingolipids and sugars. Direct injection mass spectrometric analysis was performed on an API4000 Qtrap tandem mass spectrometry instrument (Applied Biosystems/MDS Analytical Technologies, USA) equipped with a solvent delivery system. Biocrates MetIQ software (Biocrates Life Sciences AG, Austria) was used to control the assay workflow, including calculation of metabolite concentrations. A targeted profiling scheme was used to quantitatively screen for known small molecule metabolites using multiple reaction monitoring, neutral loss and precursor ion scans. For quality control standards, human plasma samples were spiked with two different concentrations of known metabolites. This procedure was performed by The Metabolomics Innovation Centre (Alberta, Canada).

### Histology and Immunohistochemistry

Knee joints were harvested at each time point and fixed for 48 h in 10% neutral buffered formalin at room temperature. Knee joints were then decalcified in rapid decalcifier solution (RDO solution; Apex Engineering Products Corporation, USA) for 1.5 h and embedded in paraffin. Sections (5 µm) were dried at 40 °C for 24 h. Each knee joint of the mouse was sectioned until the meniscus in the knee joint was separated in the anterior-posterior aspect approaching the middle of the medial portion of the knee joint. After reaching this depth, we again cut 75 more microns while collecting 5 micron serial sections. The sections were then deparaffinised, rehydrated and stained with Safranin-O/Fast green (Sigma-Aldrich, USA) according to the manufacturer’s instructions. OASRI scoring method for mouse was used to analyse and score knee joint sections by two independent, blinded reviewers (n = 10, at each time point with at least 10 sections scored per knee)^45^. To evaluate the degree of synovitis, sections were stained with Masson’s trichrome (Sigma-Aldrich, USA) as previously reported^46^. Section were then blindly scored on a scale from 0 to 3: 0, no synovitis; 1, mild synovitis; 2, moderate synovitis; and 3, severe synovitis.

Formalin-fixed, paraffin-embedded knee joint tissue sections (5 µm) from 20 week post-surgery mice were deparaffinised and treated at 60 °C for 3 h with citrate buffer, pH 6.0. Endogenous peroxidase activity was blocked with 1% H_2_O_2_ for 30 mins. Sections were blocked with 0.1% BSA for 30 mins and incubated overnight at 4 °C with Poly (ADP –ribose) polymerase (PARP) p85 (Promega, USA), leptin (Abcam, USA) (1:100) or IgG1 rabbit isotype control (Dako, Agilent Technologies, USA) in a humidified chamber. After washing twice in water, the slides were incubated with their respective biotinylated secondary antibodies for 30 mins. Signal was amplified with HRP conjugated to streptavidin using the Vectastain Elite ABC kit (Vector Laboratories, USA). Slides were developed with a DAB chromogenic substrate, including nickel, as per manufacturer’s directions (Vector Laboratories, USA) and counterstained with eosin Y (Fisher Scientific, USA). Percentage of chondrocytes per area positive to leptin and PARP p85 expression were determined (n = 5 per treatment).

### Human Specimens

Human OA cartilage was obtained from patients undergoing knee replacement (n = 10, age: 63–78 years, BMI 24.54–48.46) (Supplementary Table 3) under informed written consent. OA was diagnosed in all patients according to the American College of Rheumatology Diagnostic Criteria for OA^47^. The use of human tissue for research purposes was approved by the UHN Institutional Ethics Committee Board (REB: 14-7529AE). All the human experiment studies have been performed following relevant regulations and guidelines.

### Chondrocyte culture

Human OA chondrocytes were isolated by sequential enzymatic digestion as previously described^44^. Subconfluent cells were passaged once (P1) and seeded at a density of 3 × 10^5^ in DMEM (Invitrogen, USA) with 10% FBS (Wisent Inc., Canada) and cultured at 37 °C for about 48 h. P1 chondrocytes were cultured to 80% confluency when the media was changed to DMEM with 0.5% FBS for 24 h. Chondrocytes were then treated with vehicle (20 mM Tris-HCl pH 8.0), 500 ng/ml leptin (Sigma- Aldrich, USA), and/or 10 µM ATX inhibitor (PF-8380, Cayman Chemicals, USA) for 24 h. The concentration of leptin was selected based on previously published culture conditions^39^, reduced chondrocyte sensitivity to exogenous stimulating factors when cells are isolated from the extracellular matrix^48^, and peri-cellular concentrations likely exceeding those of circulating, physiological levels.

### ELISA and Western Blot

After treatment of P1 chondrocytes, media was collected and levels of lysophosphatidylcholine were detected using an ELISA kit for lysophosphatidylcholine (Cloud-Clone Corp, USA), as per manufacturer’s instructions. For our human chondrocyte lysoPC release assay, we measured in duplicates media from 11 wells containing cells from n = 5 independent patient samples. Following media collection, chondrocytes were washed with PBS and total protein was isolated with RIPA buffer (Sigma-Aldrich, USA) containing complete mini protease inhibitor cocktail and Phosphatase inhibitor cocktail (Roche) as per manufacturers’ instructions. Total protein was separated using Mini-PROTEAN TGX Stain-Free Gels (Bio-Rad Laboratories, Inc., USA) and transferred to Immuno-Blot PVDF Membrane (Bio-Rad Laboratories, Inc., USA) followed by blocking with 5% skimmed milk in Tris-buffered saline (TBS) for 1 h at room temperature. Blots were incubated with rabbit polyclonal antibodies specific for LOX1 (1:500) (Abcam, USA), autotaxin (1:250) (Cayman Chemicals, USA), MMP13 (1:500) (mouse monoclonal antibody, Santa Cruz Biotechnology, USA), PARPp85﻿ (1:500) (Promega, USA﻿) and β-actin (1:1000) (Sigma-Aldrich, USA) at room temperature for 1.5–2 h. Blots were washed three times with TBS + 0.1% Tween-20 (TBST) and incubated with anti-rabbit (1:10,000) or anti-mouse (1:10,000) HRP-conjugated secondary antibodies at 4 °C overnight. Signal detection was performed using enhanced chemiluminescence (ECL) reagent and the Chemidoc MP imaging system (Bio-Rad Laboratories, Inc.﻿, USA), both according to the manufacturer’s instructions. We have performed protein expression studies using cells from n = 5 patients treated with or without Leptin and n = 3 with or without ATX inhibitor. Each western blot is a representative blot from n = 3 independent experiments.

### Statistical analysis

Data are presented as mean ± SD. The data were subjected to Two-way analysis of Variance (ANOVA) followed by Holm-Sidak multiple comparison test using Prism 6 software. *P* < 0.05 was considered statistically significant. For the metabolomics study, MetaboAnalyst web-based statistical package was used^49^. Data normalizations were performed using log-transformed - auto scaling normalization. PCA analysis (principal component analysis) followed by VIP scores were calculated to identify metabolites contributing the greatest influence in discriminating individual diet groups. Metabolites with VIP scores ≥1 and showing statistical significance between groups (*p* < 0.05) by One-way ANOVA and Fisher’s LSD post-hoc tests were identified. Metabolites meeting these predefined cutoffs were plotted by mean fold change in heat maps and Venn diagrams. LD and HFD-fed mice at 9 months of age with both plasma and histologic evaluation of OA severity by OARSI scoring were subjected to univariate and multivariate ROC analysis and predictive modeling. Non-severe OA was considered as a tibial OARSI score of <3 and severe as a tibial OARSI score of ≥3. According to OARSI scoring criteria, knee joints with scores <3 have vertical clefts down to the layer immediately below the superficial layer and some loss in the surface lamina (non-severe), whereas knee joints scoring ≥3 have vertical clefts or erosion to the calcified cartilage (severe)^45^. Univariate ROC analysis and t-tests were performed on log-transformed, auto-scaled metabolite concentration data from four identified metabolites longitudinally upregulated in the plasma of HFD- fed mice, based on our described analysis above. Multivariate ROC analysis and prediction modeling was performed using a Random Forests classification method with 100 cross validations. For multivariate analysis, the AUC and predictive accuracy of the top 3 and top 4 metabolites, based on univariate analysis giving the highest area under the curve (AUC) and smallest t-test p-values (with p < 0.05 severe vs non-severe groups) were determined. Plasma samples from mice at 18-weeks of diet and 9 months of age without corresponding histological OARSI scoring data were evaluated against our model from the top 3 metabolites and probability of having severe vs. non-severe OA scores was determined.

## Electronic supplementary material

Supplementary Figure 1,Supplementary Figure 2,Supplementary Table 1, Supplementary Table 2, Supplementary Table 3
