# Supplementary material for: High-fat diet-induced acceleration of osteoarthritis is associated with a distinct and sustained plasma metabolite signature
Source: Sci Rep. 2017 Aug 15;7:8205. doi: 10.1038/s41598-017-07963-6 (PMC5557929; doi:10.1038/s41598-017-07963-6)
Supplement: Supplementary file 1 — Supplementary Figure 1,Supplementary Figure 2,Supplementary Table 1, Supplementary Table 2, Supplementary Table 3 [file 41598_2017_7963_MOESM1_ESM.pdf]

High-fat diet-induced acceleration of osteoarthritis is associated with a distinct and sustained plasma metabolite signature

Poulami Datta, Yue Zhang, Alexa Parousis, Anirudh Sharma, Evgeny Rossomacha, Helal Endisha, Brian Wu, Izabela Kacprzak, Nizar N. Mahomed, Rajiv Gandhi, Jason S. Rockel and Kapoor Mohit

Supplementary Figure 1

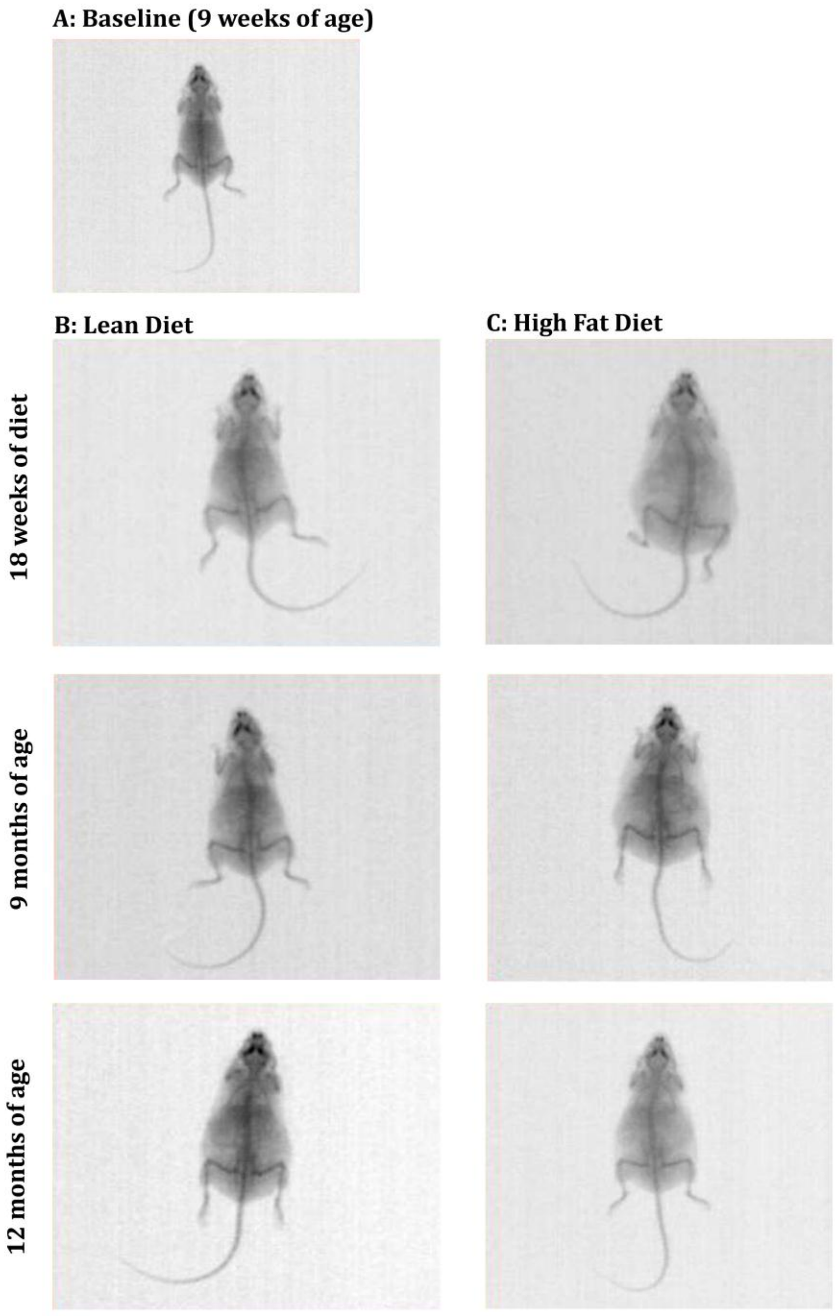

Supplementary Figure 1. Prolonged changes in body composition due to distinct diet regimes. (A) Dual energy X- ray absorptiometry (DEXA) scanning representative image of 9-week old mice prior to start of special diet regimes. (B) Representative DEXA images of lean diet-fed mice at 18 weeks of diet, 9 months of age and 12 months of age. (C) Representative

DEXA images of high fat diet-fed mice at 18 weeks of diet, 9 months of age and 12 months of age. Representative images for each diet group at each time point (n =10) per group.

Supplementary Figure 2

A

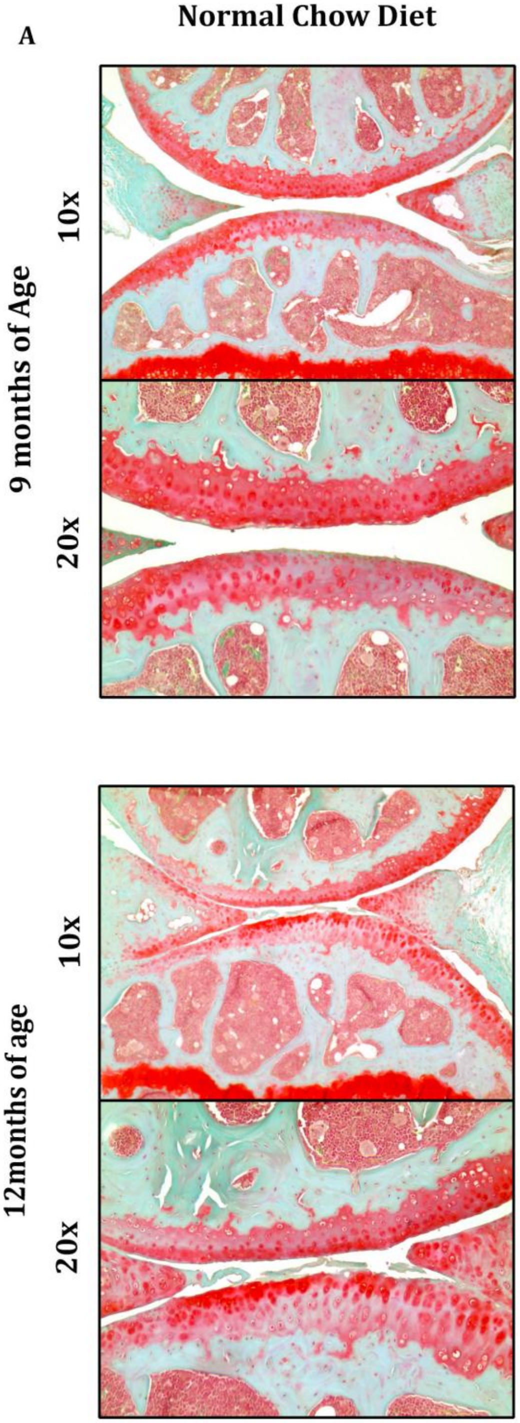

B

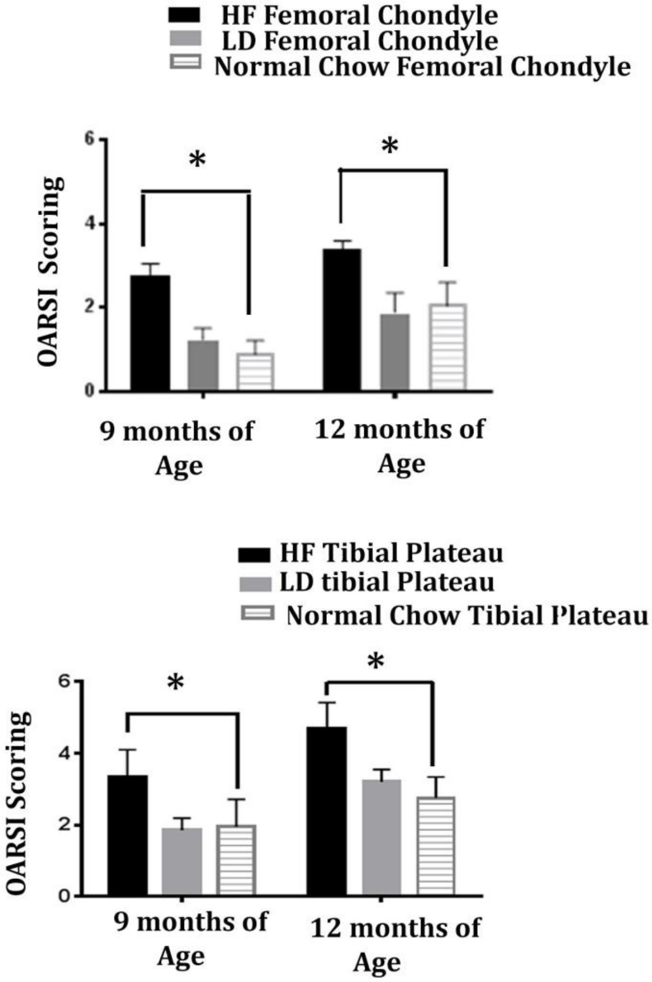

C

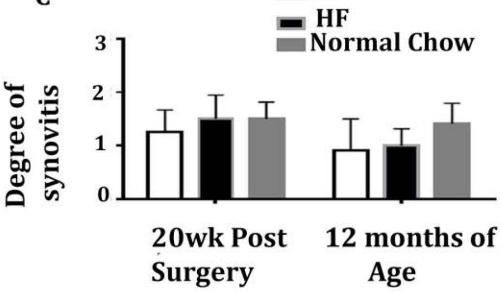

Supplementary Figure 2. Normal chow diet fed mice showed similar OA characteristics as compared to LD- fed mice. (A) Representative histological sections of SafO stained knee joints from normal chow diet-fed mice at 9 months and 12 months of age are shown. Magnifications: 10x and 20x. (B) Severity of OA pathogenesis was determined by OARSI histopathology grading of mouse medial femoral condyles and medial tibial plateaus. Data are expressed as mean ± SD. *$p < 0.05$; n=5. Data was analysed by Two-way ANOVA followed by Holm-Sidak multiple comparison tests. *$p < 0.05$ HFD at 9 months of age vs normal chow diet at 9 months of age and HFD at 12 months of age vs. Normal chow diet at 12 months of age (n=10 Vs n=5, n=10 Vs n=5). (C) Degree of synovitis between HFD-fed mice at 20 weeks post-surgery, LD-fed mice and normal chow diet-fed mice at 20 weeks post-surgery showed no significant difference. Similarly no significant differences in synovial inflammation between HFD-, LD- and normal chow diet-fed mice at 12 months of age were found. Data were analysed by Two-way ANOVA.

Supplementary Table 1. Sample data used to generate random forests classification models. Classes were separated based on OARSI scoring of the Tibia. Severe OARSI ≥ 3. Non-severe OARSI < 3. Metabolite biomarker concentrations (pg/ml) used for model generation are listed. 9M, 9 months; LD, Lean diet; HFD, High fat diet. OARSI, osteoarthritis research society international; lysoPC, lysophosphatidylcholine; PC, Phosphatidylcholine. Grey highlighted samples are classified to the wrong groups based on the model outcome.

**Name**	**OARSI Score**	**Class**	**lysoPCaC17:0**	**lysoPCaC18:0**	**lysoPCaC20:4**	**PCaaC36:2**
14RF_9M_LD	F: 2.5; T: 2.5	non-severe	3.65	67.71	42.46	280.57
13NP_9M_LD	F: 2.5; T: 2.0	non-severe	3.30	70.47	33.98	326.61
13LF_9M_LD	F: 2; T:2	non-severe	3.74	74.81	38.07	351.89
8LF_9M_LD	F:1.5; T: 1.5	non-severe	3.98	77.46	47.12	346.60
7RF_9M_LD	F:3; T: 2	non-severe	4.49	79.08	45.00	402.53
13RF_9M_LD	T: 2; T:2	non-severe	3.97	89.32	44.47	371.44
14NP_9M_LD	T:2; T:2	non-severe	3.47	92.17	52.41	334.74
1LB_9M_HFD	F: 4; T:3	severe	4.15	79.79	45.05	420.52
16LF_9M_HFD	F:3.5;T:5	severe	5.43	84.56	66.82	368.44
16LB_9M_HFD	F:4 T: 3	severe	5.32	113.43	84.76	439.82
16RF_9M_HFD	F:5; T:3.5	severe	6.62	129.68	88.54	428.47

Supplementary Table 2. Predicted classification of samples used for the identification of longitudinally changed metabolites that did not have matching OARSI scoring, as determined by the classification model generated from Top 3 Metabolites used as biomarkers. Metabolite biomarker concentrations (pg/ml) used for model generation are listed. Grey highlighted samples are predicted to be classified to the wrong groups based on known diet. 18W, 18 weeks; 9M, 9 months; LD, Lean diet; HFD, High fat diet. OARSI, osteoarthritis research society international; lysoPC, lysophosphatidylcholine; PC, Phosphatidylcholine. Grey highlighted samples are classified to the wrong groups based on the model outcome.

Name	Probability	Class	lysoPCaC17:0	lysoPCaC18:0	lysoPCaC20:4	PCaaC36:2
11LF_18W_LD	1.00	non-severe	2.21	32.34	15.44	268.45
12RB_18W_LD	1.00	non-severe	2.24	35.64	16.30	264.77
12LF_18W_LD	1.00	non-severe	2.66	44.81	27.25	283.33
10RF_18W_LD	1.00	non-severe	3.31	55.64	22.93	313.36
7RF_18W_LD	1.00	non-severe	3.66	70.92	43.39	291.36
7RB_18W_LD	1.00	non-severe	3.55	70.50	43.92	269.71
8NP_18W_HFD	0.99	severe	6.02	134.58	82.00	517.22
7LF_18W_HFD	0.99	severe	8.76	159.82	90.65	605.21
9NP_18W_HFD	0.99	severe	9.07	180.25	70.42	717.62
9LF_18W_HFD	0.99	severe	9.17	181.27	82.38	720.56

8LB_18W_HFD	0.99	severe	7.63	166.28	99.57	725.13
7RB_18W_HFD	0.99	severe	8.10	183.00	109.31	525.72
9RB_18W_HFD	0.99	severe	11.52	227.58	85.84	910.06
8RF_9M_LD	0.96	non-severe	3.96	78.86	36.06	386.31
19LF_18W_HFD	0.95	severe	6.07	117.40	65.10	535.69
19RF_18W_HFD	0.93	severe	5.11	93.97	66.39	537.28
7LF_9M_LD	0.92	non-severe	3.71	62.21	56.72	321.68
19NP_18W_HFD	0.89	severe	6.94	127.35	56.50	536.68
1RF_9M_HFD	0.84	severe	5.29	102.79	53.17	469.82
8RF_18W_LD	0.83	non-severe	4.82	81.13	37.73	314.74
14RF_18W_LD	0.82	non-severe	3.26	78.56	61.56	302.32
12LF_9M_HFD	0.77	non-severe	3.53	69.67	61.53	313.27
11LB_18W_LD	0.67	non-severe	4.15	99.06	54.55	378.75
9LF_9M_HFD	0.63	severe	6.41	91.78	47.62	403.93

7RB_9M_LD	0.60	non-severe	4.91	81.16	51.28	342.10
2LF_9M_HFD	0.58	severe	4.76	89.18	62.71	374.12
9RF_9M_HFD	0.58	severe	4.63	96.63	60.86	377.63
2RB_9M_HFD	0.55	non-severe	4.52	94.93	61.63	339.50
13RF_18W_LD	0.53	non-severe	4.10	94.41	70.00	296.77

Supplementary Table 3: Characteristics of human OA chondrocytes used for cell culture studies**:**

OA Cartilage	Age	Sex	BMI (at time of Surgery)
Cartilage Specimen 1	74	F	48.46
Cartilage Specimen 2	69	F	28.43
Cartilage Specimen 3	77	M	33.69
Cartilage Specimen 4	78	M	26.96
Cartilage Specimen 5	65	M	38.30
Cartilage Specimen 6	63	M	24.54
Cartilage Specimen 7	77	M	29.76
Cartilage Specimen 8	71	F	26.50
Cartilage Specimen 9	61	M	37.72
Cartilage Specimen10	63	F	36.98
